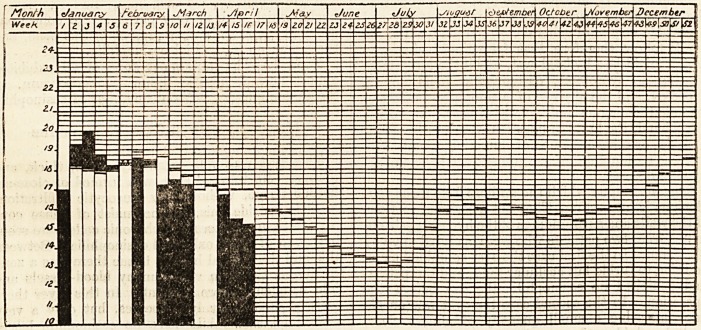# Diagram of the Weekly Death Rate in 1908

**Published:** 1908-05-02

**Authors:** 


### Public Health and Hygiene.

### DIAGRAM OF THE WEEKLY DEATH RATE IN 1908.

Showing the weekly death rate for 1908 according to the Registrar-General and the mean weekly death rate for the last six years of the 76 great towns of England and Wales.

White columns show mean weekly death rate for last six years. Black columns show weekly death rate for current year. Where death rate for 1908 is in excess of the six yearly mean the excess is shown in black above the white column, which represents the mean.

Where death rate for 1908 is below the six yearly mean the black column is shown in its entire length, the white column, which represents the mean, showing above the black.

Where the death rate for 1908 coincides with the six yearly mean, it is shown thus XX.

## Figures and Tables

**Figure f1:**